# Robust backup support technique with dual guide extension catheters: a case report

**DOI:** 10.1093/ehjcr/ytaf412

**Published:** 2025-08-22

**Authors:** Kuniyasu Ikeoka, Yasunori Ueda, Koichi Inoue, Yasushi Matsumura

**Affiliations:** Cardiovascular Division, National Hospital Organization Osaka National Hospital, 2-1-14 Hoenzaka, Chuo-ku, Osaka 540-0006, Japan; Cardiovascular Division, National Hospital Organization Osaka National Hospital, 2-1-14 Hoenzaka, Chuo-ku, Osaka 540-0006, Japan; Cardiovascular Division, National Hospital Organization Osaka National Hospital, 2-1-14 Hoenzaka, Chuo-ku, Osaka 540-0006, Japan; Cardiovascular Division, National Hospital Organization Osaka National Hospital, 2-1-14 Hoenzaka, Chuo-ku, Osaka 540-0006, Japan

**Keywords:** Percutaneous coronary intervention, Chronic total occlusion, Guide extension catheter, Backup force, Case report

## Abstract

**Background:**

Guide extension catheters are specially designed for percutaneous coronary intervention (PCI) to enhance backup support of the guide catheter by providing coaxial alignment, thereby allowing deep intubation of the catheter. We have developed an innovative auxiliary support technique utilizing a dual guide extension catheter system, designed to enhance safety and facilitate deep coronary artery access.

**Case summary:**

A male in his sixties who presented with chest pain was diagnosed with non-ST elevation myocardial infarction. Percutaneous coronary intervention failed to achieve complete revascularization due to the presence of extensive chronic total occlusion (CTO) in the right coronary artery (RCA). A staged percutaneous intervention was performed to treat the tortuous CTO lesion. Dual guide extension catheters (DGEC), consisting of a 6 Fr Guideplus™ in the 8 Fr GuideZilla II™, were introduced. The Guideplus™ was deeply inserted into the distal RCA through the GuideZilla II™ support. This system provided a stable catheter platform, enabling successful negotiation of the guidewire and delivery of the catheter across the extensive, markedly tortuous segment of CTO of the RCA. Thereafter, the RCA was completely recanalized through stenting and balloon angioplasty following antegrade guidewire penetration. Subsequently, enhanced backup force of the DGEC was demonstrated through *in vitro* force gauge testing, suggesting that this novel technique might be promising for interventions for CTO in vessels with severe tortuosity.

**Discussion:**

The DGEC system facilitates substantial guide catheter backup support in the context of PCI for severely tortuous total occlusive disease.

Learning points

- The dual guide extension catheter (DGEC) system enhances catheter backup support in percutaneous coronary intervention, facilitating device deliverability through tortuous chronic total occlusion lesions.
- The DGEC system is designed with a leading, flexible, soft-tipped, inner guide extension catheter that enables its deep intubation into the vessel.

## Introduction

Advances in device development have led to the evolution of the procedure of percutaneous coronary intervention (PCI). However, deliverability of catheter is still one of the common challenges faced by interventional cardiologists. In particular, inadequate guide catheter backup force in tortuous anatomy frequently leads to failure of device delivery and guidewire penetration, especially in right coronary artery (RCA) interventions.

One of the most widely used techniques for guide catheter support is the use of guide extension catheters.^1^ This technology was first introduced in clinical practice in 2009.^2^ Guide extension catheters are now considered essential equipment for PCI due to their ability to enhance backup support of the guide catheter by providing coaxial alignment, allowing deep insertion of the catheter.^3^ However, their efficacy remains limited in cases with extreme vascular tortuosity, where the loss of translational force through multiple bends prevents effective force transmission to the distal lesion.

The ‘Mother and Child’ technique was developed to improve support for coronary interventions. In this technique, longer, flexible, and lower-profile guide extension catheters are advanced over the conventional guide catheters and are extended beyond the distal tip of the guide catheter to enable access to deeper vessels, consequently achieving enhanced support and coaxial alignment.^4^

We developed an innovative auxiliary support technique utilizing a third-generation guide catheter system. This dual guide extension catheter (DGEC) system is designed to facilitate easy, safe, and deep coronary access. We demonstrate its application in a case of RCA-CTO with severe tortuosity, where conventional methods failed. Furthermore, we provide quantitative bench test evidence comparing the DGEC system’s mechanical performance with traditional single-extension approaches.

## Summary figure

**Figure:**
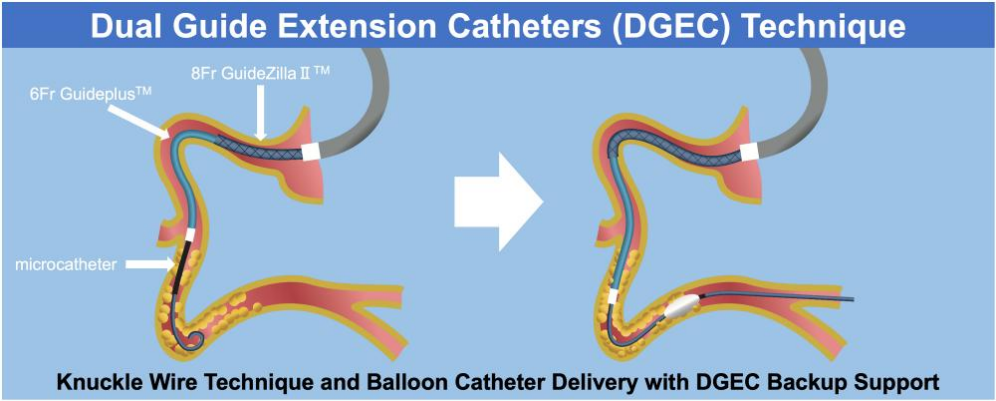


## Case presentation

A 64-year-old male with a history of hypertension, dyslipidaemia, and hyperuricaemia presented with sudden onset of chest tightness. Three days later, he was diagnosed with non-ST elevation myocardial infarction at a regional hospital and was transferred to our hospital for intensive care treatment. His electrocardiogram showed abnormal Q waves in leads III and aVF, and echocardiography showed severe hypokinesis of the infero-posterior wall at the cardiac base. Blood tests revealed significant elevation of troponin T to 1521 ng/mL.

**Figure 1:**
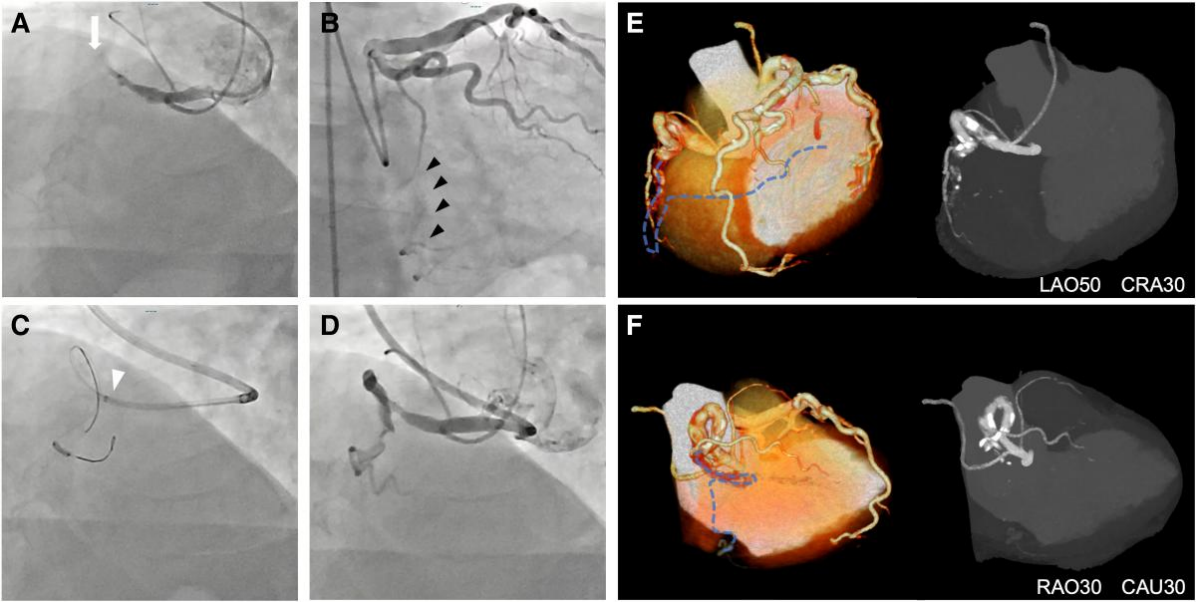
Coronary angiography and cardiac computed tomography angiography of this case. (*A*) Emergency angiography demonstrated thromboembolic occlusion of the right coronary artery (*white arrow*). (*B*) The atrial branch of the circumflex artery supplied the distal right coronary artery via a severely tortuous collateral artery (*black arrowheads*). This collateral artery could not be used for a retrograde approach. (*C*) The side branch balloon anchoring technique was unsuccessful for guidewire penetration of the chronic total occlusion due to insufficient backup force (*white arrowhead*). (*D*) The proximal right coronary artery was recanalized by thrombectomy. (*E* and *F*) Computed tomography angiography demonstrated the long chronic total occlusion lesion with sparse mural calcification. The dotted blue line represents the estimated vessel course, showing a severely tortuous occluded lesion in the distal right coronary artery segment.

Coronary angiography revealed a thromboembolic occlusion in the RCA (*Figure 1A*), with tortuous collateral vessels from the circumflex artery (LCx) in the atrioventricular groove (*Figure 1B*). Subsequently, coronary revascularization was attempted via a transfemoral approach. However, we were unable to engage the RCA despite using guide catheters of various shapes due to significant thoracic and abdominal aortic tortuosity. Only a hockey stick–shaped guide catheter could selectively hook the ostium, resulting in inadequate catheter backup. Hence, a 7 Fr GuideZilla II™ (Boston Scientific, MA, USA) guide extension catheter was introduced to strengthen and back up the catheter. This allowed successful performance of balloon angioplasty and catheter thrombectomy in the proximal RCA. However, complete revascularization failed due to the presence of extensive CTO of the distal RCA. Additionally, the side branch balloon anchoring technique could not successfully achieve guidewire penetration of the CTO due to insufficient backup force (*Figure 1C* and *D*). Finally, the first intervention session resulted in reperfusion of the right ventricular branch via the proximal RCA.

**Figure 2:**
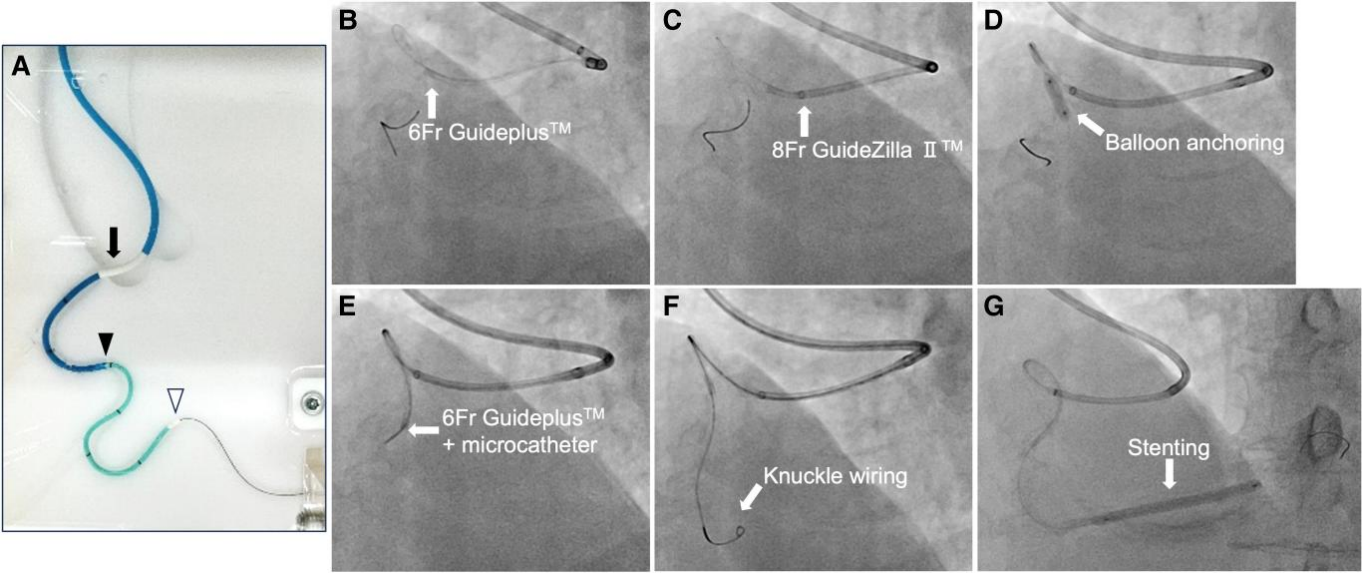
Procedural flow of the dual guide extension catheter system for right coronary intervention. (*A*) The dual guide extension catheter system in the artificial tortuous coronary vessel model with the 8 Fr guide catheter (*black arrow*). The outer 8 Fr guide extension catheter (GuideZilla II^™^: *black arrowhead*) and the inner soft-tipped 6 Fr guide extension catheter (Guideplus^™^: *white arrowhead*) were deeply inserted into the vessel model. (*B–G*) The dual guide extension catheter procedure in our patient. The soft-tipped 6 Fr guide extension catheter (Guideplus^™^) was inserted into the vessel after guidewire passage (*B*). The outer 8 Fr guide extension catheter (GuideZilla II^™^) was inserted over the 6 Fr guide extension catheter (*C*). The 6 Fr guide extension catheter was advanced utilizing the balloon anchoring technique and was deeply inserted into the distal portion of the vessel with outer 8 Fr guide extension catheter support (*D* and *E*). This dual guide extension catheter system provided a stable catheter platform, facilitating the knuckle wire procedure with the Gladius MG14 guidewire (*F*). The dual guide extension catheter system enabled delivery of the stent system into the distal right coronary artery through the severely tortuous vessel segment (*G*).

Following this procedure, cardiac computed tomography (CT) was performed, which demonstrated marked tortuosity in the RCA, characterized by two 90-degree bends and two looped bends (*Figure 1E* and *F*). Mural calcification was too sparse to identify the coronary path. A staged intervention was performed to treat the CTO via the transfemoral approach utilizing the same 8 Fr hockey stick guide catheter. Since intervention via the retrograde approach was not feasible due to tortuous collaterals from the LCx via the atrioventricular groove, we focused on antegrade wire negotiation. In order to gain additional backup force, the DGEC system, consisting of the 6 Fr Guideplus™ (NIPRO, Tokyo, Japan) in the 8 Fr GuideZilla II^™^, was introduced (*Figure 2A*). The procedural steps of the DGEC system were as follows: an 8 Fr guide extension catheter was inserted into the vessel after guidewire passage. The 6 Fr guide extension catheter was inserted using the balloon anchoring technique and was deeply positioned in the distal portion of the vessel with outer guide extension catheter support (*Figure 2B–E*). The DGEC system provided a stable catheter platform, facilitating successful negotiation of a 3 g tip load guidewire. The knuckle wire technique using the Gladius MG14 guidewire (Asahi Intecc, Aichi, Japan) was able to successfully penetrate the distal true lumen (*Figure 2F*). The DGEC system enabled delivery of the stent system into the distal RCA through the severely tortuous bent tract (*Figure 2G*), with subsequent complete recanalization by stenting and balloon angioplasty (Supplementary material online, *Video S1*). The total procedural time was 290 min with a contrast volume of 125 mL. The patient’s renal function remained normal after the procedure.

**Figure 3:**
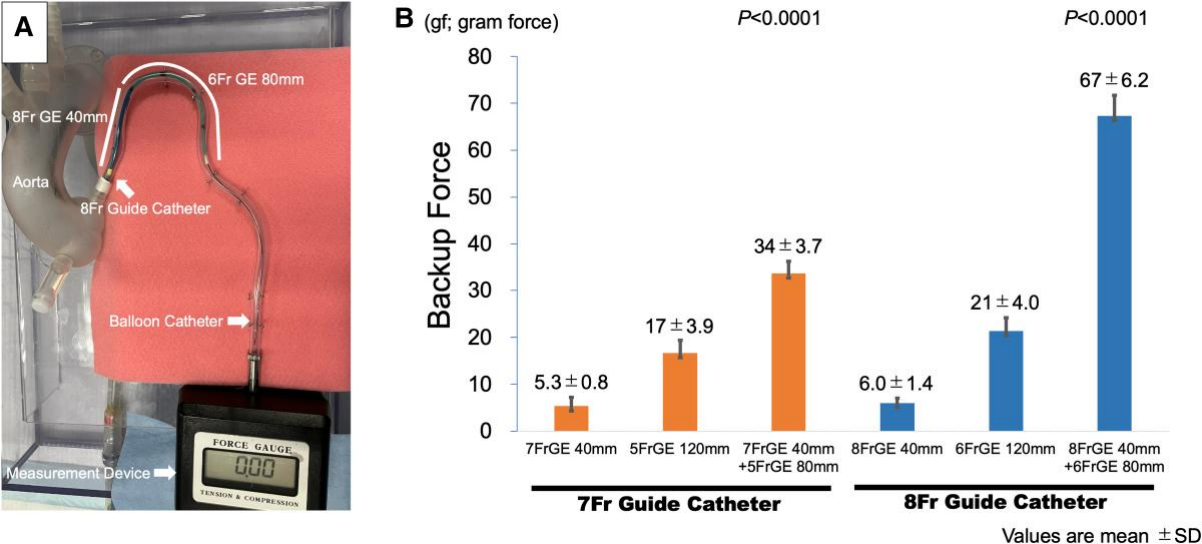
Experimental coronary model for measurement of the backup force. (*A*) We assessed the maximal backup force of dual guide extension catheter systems using 7Fr and 8Fr guiding catheters in the artificial tortuous coronary vessel model of transfemoral intervention. (*B*) The bench test revealed significant advantage of the dual guide extension catheter system over the single guide extension system. GE, guide extension catheter.

Subsequently, we performed an *in vitro* comparison of the backup force of the DGEC system with the conventional single guide extension method in a bench test (*Figure 3A*). The measurement instrument used to assess the guide catheter backup force was an artificial tortuous coronary vessel model of transfemoral intervention, consisting of a 3D-printed artificial aortic arch and bent plastic tube. A guidewire was passed through the guide catheter tip placed at the artificial coronary ostium into the plastic tube. Then, the guidewire was captured by a guidewire torque device connected to a force gauge. We defined the maximal backup force when the guide catheter disengaged by pushing the torque device with the balloon catheter. We used this method to measure both, an 8 Fr and a 7 Fr DGEC system. The 7 Fr DGEC system consisted of a 5 Fr guide extension catheter in a 7 Fr guide extension catheter. The bench test revealed considerable superiority of the DGEC system over the single guide extension system (*Figure 3B*), suggesting that the novel DGEC technique is promising for interventions in vessels with severe tortuosity in the context of CTO.

**Figure 4:**
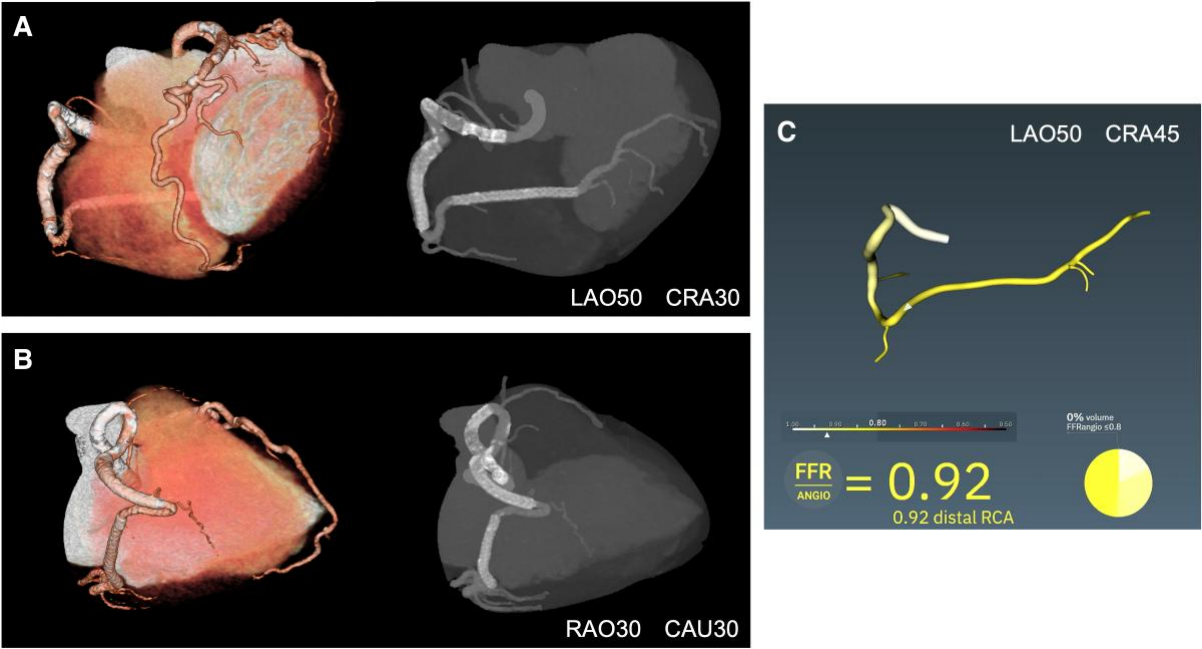
Follow-up computed tomography and angiography-based fractional flow reserve. (*A* and *B*) Follow-up computed tomography imaging at 6 months demonstrated excellent coronary artery patency. The mildly curved segments were treated with drug-eluting stents, and severely tortuous segments were treated with drug coated balloons. (*C*) Angiography-based fractional flow reserve revealed substantial coronary blood flow at 12 months after percutaneous coronary intervention.

Follow-up CT imaging at 6 months in this patient demonstrated excellent coronary artery patency (*Figure 4A* and *B*, Supplementary material online, *Video S2*). Coronary angiography at 12 months also showed sustained patency in the RCA. Subsequently, physiological examination by angiography-based fractional flow reserve revealed substantial coronary blood flow (*Figure 4C*).

## Discussion

We described here the use of a novel technique utilizing a DGEC to obtain extra backup force in a case with an exceptionally tortuous right coronary vessel. Guide catheters with a single guide extension catheter are limited in their ability to establish robust backup systems, due to the inability of deep intubation with the guide extension catheter. We developed a system for deep intubation of the catheter utilizing a soft-tipped thin guide extension catheter with a large guide extension support. A tortuous vessel model bench test clarified the robust backup force with this DGEC system. Only soft-tipped Guideplus™ guide extension catheters are feasible for inner guide extension.^5^ Other products, such as GuideZilla II™ and GuideLiner™ are not suitable for inner guide extension, because their catheter tips are too hard to insert into the proximal orifice of large guide extension catheter tubes.

Tanaka *et al*.^6^ reported that the locking method achieved the highest backup force among the Mother and Child method and the anchoring balloon technique. In our case, the locking method was less feasible due to serious vessel tortuosity and the large vessel diameter. Although we tried the balloon anchoring technique at the first session, the method did not work due to lack of substantial backup force. The DGEC system enables deep catheter intubation by using a supportive large bore guide extension catheter along with a soft-tipped guide extension catheter. This dual deep insertion system provides a robust backup force in the seriously tortuous vessels.

The CrossLiner™ (Teleflex, NC, USA) is a next-generation guide extension system intended to allow deep insertion of catheters into the coronary artery.^7^ Generally, delivery of the guide extension over a balloon catheter shaft is sometimes dangerous, because the blunt edged tubular structure can chip vessel plaques or calcium and can get stuck in stent struts. The CrossLiner™ is specifically designed with a leading, flexible, low profile, monorail, inner microcatheter that enables its deep insertion into the vessel. The DGEC system also comprises of a soft-tipped guide extension catheter (Guideplus™) as the leading inner guide extension. These two catheter systems have similar features focusing on deep insertion.

## Conclusion

The DGEC method facilitates substantial guide catheter support in the context of coronary revascularization of severely tortuous occlusive disease.

## Supplementary Material

ytaf412_Supplementary_Data

## Data Availability

The data underlying this article are available in the article.

## Lead author biography

Dr Kuniyasu Ikeoka serves as an interventional specialist at NHO Osaka National Hospital, Osaka, Japan. He obtained his medical degree from Medical School of Osaka University, Osaka, Japan. Subsequently, he pursued his post-graduate training at Kansai Rosai Hospital, Hyogo, Japan. He completed a doctoral programme in medical science at Osaka University. His main interests are innovative transcatheter vascular therapy and advanced vascular imaging techniques.

## Supplementary material

**Consent:** The authors confirm that written informed consent was obtained from the patient for the publication of this case report and any accompanying images. The authors affirm that this report complies with the ethical standards of the Committee on Publication Ethics (COPE).

**Funding:** None declared.
